# Association Between N363S and BclI Polymorphisms of the Glucocorticoid Receptor Gene (NR3C1) and Glucocorticoid Side Effects During Childhood Acute Lymphoblastic Leukemia Treatment

**DOI:** 10.4274/tjh.2016.0253

**Published:** 2017-06-01

**Authors:** Meriç Kaymak Cihan, Halil Gürhan Karabulut, Nüket Yürür Kutlay, Hatice Ilgın Ruhi, Ajlan Tükün, Lale Olcay

**Affiliations:** 1 Dr. Abdurrahman Yurtaslan Ankara Oncology Training and Research Hospital, Clinic of Pediatrics, Division of Pediatric Hematology-Oncology, Ankara, Turkey; 2 Ankara University Faculty of Medicine, Department of Medical Genetics, Ankara, Turkey

**Keywords:** Acute lymphoblastic leukemia, Glucocorticoid receptor gene, BclI and N363S polymorphisms

## Abstract

**Objective:**

Glucocorticoids (GCs) are the key drugs for the treatment of pediatric acute lymphoblastic leukemia (ALL). Herein, investigation of the relationship between the N363S and BclI polymorphisms of the GC receptor gene (*NR3C1*) and the side effects of GCs during pediatric ALL therapy was aimed.

**Materials and Methods:**

N363S and BclI polymorphisms were analyzed in 49 patients with ALL treated between 2000 and 2012. The control group consisted of 46 patients with benign disorders. The side effects of GCs noted during the induction and reinduction periods were evaluated retrospectively according to the National Cancer Institute’s Common Terminology Criteria for Adverse Events, version 4.0.

**Results:**

The BclI allele and genotype frequencies were found similar in the two groups. No N363S polymorphism was detected in either of the groups. During induction, dyspepsia was found more frequently in the CG than in the CC (wild-type) genotype (36.4% vs. 5.3%, p=0.018) and depression symptoms more frequent in patients with the G allele (CG+GG) than the CC genotype (39.3% vs. 10.5%, p=0.031). During reinduction, Cushingoid changes, dyspepsia, and depression symptoms were more frequent in patients with the G allele (CG+GG) than in patients with the CC genotype (48.1% vs. 17.6%, p=0.041; 29.6% vs. 0.0%, p=0.016; 40.7% vs. 11.8%, p=0.040, respectively).

**Conclusion:**

In our study, patients with the BclI polymorphism were found to have developed more frequent side effects. We think that the BclI polymorphism should be considered while designing individualized therapies in childhood ALL.

## Abstract (tr)

**Amaç:**

Çocukluk çağı akut lenfoblastik lösemisinde (ALL), glukokortikoidler (GC) tedavinin ana yapı taşını oluşturmaktadırlar. Bu çalışmada pediatrik ALL tedavisi sırasında GC’lerin yan etkileri ile GC reseptör geninin (*NR3C1*) N363S ve BclI polimorfizmleri arasındaki ilişkinin araştırılması amaçlandı.

**Gereç ve Yöntemler:**

N363S ve BclI polimorfizmleri 2000 ile 2012 yılları arasında tedavi edilmiş 49 ALL hastasında incelendi. Kontrol grubu benign hastalıkları olan 46 çocuktan oluşturuldu. İndüksiyon ve reindüksiyon sırasında görülen GC yan etkileri Ulusal Kanser Enstitüsü’nün “Advers Etkiler için Ortak Terminoloji Kriterleri” (CTCAE version 4,0), kullanılarak dosya ve hemşire gözlemlerinden geriye dönük olarak incelendi.

**Bulgular:**

BclI alel ve genotip sıklığı açısından iki grup arasında fark yoktu. N363S polimorfizmi her iki grupta da tespit edilmedi. İndüksiyon sırasında, dispeptik yakınmalar CG genotipinde CC (yaban tip) genotipine göre daha sık görüldü (%36,4-%5,3; p=0,018) ve depresyon semptomları G alelini taşıyanlarda (CG+GG), CC genotipine göre daha sık tespit edildi (%39,3-%10,5; p=0,031). Reindüksiyon sırasında, Cushingoid değişiklikler, dispeptik yakınmalar ve depresyon semptomları G aleli taşıyanlarda (CG+GG) CC genotipine göre daha fazla tespit edildi (sırasıyla %48,1-%17,6, p=0,041; %29,6-%0,0 p=0,016; %40,7-%11,8; p=0,040).

**Sonuç:**

Çalışmamızda, BclI polimorfizmine sahip bireyler daha sık yan etki gösterdiler. Çocukluk çağı ALL tedavisinde bireysel tedaviler geliştirilirken BclI polimorfizminin de göz önünde bulundurulmasının gerektiğini düşünüyoruz.

## INTRODUCTION

Glucocorticoids (GCs) are key drugs for the treatment of pediatric acute lymphoblastic leukemia (ALL) [1,2]. Their antileukemic effects occur through the induction of apoptosis and/or cell cycle arrest [3]. However, they give rise to severe side effects, which also show individual variation. Therefore, in this study we focused on individual genetic differences that may lead to increased sensitivity to GCs and their side effects.

GC downstream effects are mediated through the process of binding to an intracytoplasmic glucocorticoid receptor (GR). This receptor gene (*NR3C1*) is located on the long arm of the fifth chromosome (5q31.3). A number of GR polymorphisms have been detected in recent years [4,5]. The 1220A/G transition that results in the substitution of asparagine to serine at codon 363 (N363S polymorphism) and the substitution of cytosine (C) to guanine (G) at intron 2 (BclI polymorphism) are related to high sensitivity to GCs [6]. The C allele is the most frequently occurring and thus can be considered the wild-type allele [7]. These polymorphisms cause personal variability in the sensitivity and responses to GCs [8]. In a study by Huizenga et al. [5], the carriers of the N363S polymorphism had lower cortisol levels after low-dose dexamethasone (0.25 mg) suppression tests. Cuzzoni et al. [6] showed that the BclI polymorphism caused increased sensitivity to methylprednisolone in human lymphocytes in vitro. Lin et al. [9] found that the frequency of N363S allele carriers in subjects with coronary artery disease was particularly high. Di Blasio et al. [10] found that among obese patients, heterozygous N363S carriers had significantly higher body mass index (BMI) when compared to wild-type homozygotes. The BclI polymorphism also plays an important role in certain psychiatric diseases [11,12,13]. The mechanism of GC sensitivity is still incompletely understood but the hypothesis that the BclI polymorphism changes GR dimerization and interactions between transcription factors is suggested [14].

There is variability in the treatment responses and side effects in patients with ALL [15,16], including GC treatment. While there have been a number of investigations of the GC response and GC receptor mutations and polymorphisms [3,17,18,19,20,21], there are a limited number of studies about the side effects of GCs in relation to GC receptor polymorphisms [22,23,24,25,26]. We hypothesized that the N363S and BclI polymorphisms could be important in the variability of side effects of GCs among patients with ALL. We think that this information will be important for improving personal treatment modalities in the future.

## MATERIALS AND METHODS

### Study Population

The necessary sample size was calculated to be at least 40 individuals in each group, with 80% power and 0.05 type I error while anticipating a deviation of ±20% in the control group (R 3.0.1. open source program), based on the literature data [20]. Accordingly, a total of 49 patients (age: 1.4-17 years; 31 males, 18 females), 14 and 35 of whom were treated with the St Jude TXIII and Berlin-Frankfurt-Münster (BFM)-TR ALL 2000 protocols (the latter is modified from BFM 95) respectively between 2000 and 2012, were included in the study. Each patient enrolled in this study received GCs during the induction and reinduction phases. The patients received steroids as prednisolone at a daily dosage of 40 mg/m^2^ between day 1 and day 29 during the induction and reinduction phases in the St Jude TXIII protocol [2]. The types of steroids administered during the BFM-TR ALL 2000 protocol were prednisolone at 60 mg/m^2^/day for 31 days in high-risk and 36 days in standard-risk and intermediate-risk patients during induction (protocol I/phase I) and dexamethasone at 10 mg/m^2^/day during protocol II/phase I (reinduction). Forty-six patients aged 1.2-17.5 years with immune thrombocytopenia, nutritional anemia, or acute infections were included in the control group.

### Analysis of Glucocorticoid-Induced Side Effects

We collected clinical and laboratory data retrospectively from patient files and classified potential side effects of GCs according to the National Cancer Institute’s Common Terminology Criteria for Adverse Events, version 4.0 (CTCAE v.4) [27], which were defined as follows:

**Metabolic and Nutritional Disorders:** Hyperglycemia (fasting glucose value >100 mg/dL), hypertriglyceridemia (blood triglyceride value >150 mg/dL), obesity (BMI >25).

**Endocrine Disorders:** Cushingoid changes (buffalo hump obesity, striations, adiposity, hypertension, diabetes, and osteoporosis).

**Gastrointestinal and Hepatic Disorders:** Dyspepsia (an uncomfortable and often painful feeling in the stomach, burning stomach, bloating, heartburn, nausea, and vomiting), oral mucositis, gastric hemorrhage, and elevation in alanine aminotransferase, aspartate transaminase, and blood bilirubin levels.

**Musculoskeletal and Connective Tissue Disorders:** Arthralgia, arthritis, generalized muscle weakness, myalgia, avascular necrosis, osteoporosis. Hip joint magnetic resonance imaging (MRI) was performed for detection of avascular necrosis if the patient had hip pain during or at the end of the induction and reinduction phases. Bone mineral densitometry (BMD) for osteoporosis was performed at the time of diagnosis and at the end of the induction and reinduction phases. At the end of induction 32 patients and at the end of reinduction 34 patients were investigated for BMD. Z-scores (corrected for age) between -1 and -2.5 and Z-scores below -2.5 were considered “osteopenia” and “osteoporosis,” respectively.

**Psychiatric Disorders:** Depression symptoms (melancholic feelings of grief or unhappiness, change in sleep patterns, pessimistic thoughts, thoughts of death).

**Nervous System Disorders:** Leukoencephalopathy.

**Eye Disorders:** Blurred vision, cataract, glaucoma.

**Cardiac Disorders:** Sinus bradycardia, sinus tachycardia.

**Vascular Disorders:** Hypertension, thrombosis (superficial and deep vein).

**Infections:** Number of febrile neutropenia (FEN) attacks.

### Genetic Analysis

Peripheral blood samples were collected from all patients when they were in remission. Genomic DNA isolation was performed using the standard salting-out method. N363S and BclI genotypes were determined by polymerase chain reaction (PCR)-restriction fragment length polymorphism. PCR was performed in total volumes of 20 µL containing 0.1 µg of genomic DNA, 10 pmol of each primer (forward: 5’-CCAGTAATGTAACACTGCCCC-3’, reverse: 5’-TTCGACCAGGGGAAGTTCAGA-3’ for N363S and forward: 5’-GAGAAATTCACCCCTACCAAC-3’, reverse: 5’-AGAGCCCTATTCTTCAAACTG-3’ for BclI), 0.2 mM of each dNTP, 10 mM Tris, 50 mM KCl, 1.5 mM MgCl2, and 1 U of Taq polymerase. PCR conditions were as follows: initial denaturation at 95 °C for 4 min and then 30 cycles of 95 °C for 1 min, 56 °C for 1 min, and 72 °C for 1 min, followed by a final extension at 72 °C for 7 min. Restriction digestion was performed in total volumes of 20 µL containing 10 µL of PCR product and 2 U of restriction enzyme (Tsp5091 for N363S and BclI for BclI). Reaction mixtures were incubated overnight at 65 °C for Tsp5091 and at 55 °C for BclI. Genotypes were determined by 2% agarose gel electrophoresis of restriction digests. In the absence of the N363S polymorphism, a 357-bp PCR product gave restriction fragments of 135, 73, 70, 60, and 19 bp, whereas the polymorphic allele produced four fragments of 135, 92, 70, and 60 bp. For the BclI polymorphism, 418-bp PCR products gave two fragments of 263 and 151 bp, whereas the polymorphic allele remained uncut.

### Statistical Analysis

Data analysis was done using SPSS 11.5 for Windows. The Kolmogorov-Smirnov test was used to investigate whether the distribution of discrete numerical variables was near normal. Descriptive statistics for discrete numerical variables were shown as a median (minimum-maximum). The number of cases and the categorical variables were shown as frequency percentage (%).

For comparison of the groups, the Mann-Whitney U test was used when the number of independent groups was 2 and the Kruskal-Wallis test was used when the number was >2.

We tested for Hardy-Weinberg equilibrium for allele and genotype frequencies and they were found to be in equilibrium in both the patient and control groups.

Categorical variables were investigated by Pearson chi-square or Fisher exact test. For the comparison of allele and genotype frequencies of the control and patient groups and for comparison of side effect frequencies between the genotypes we used the Pearson chi-square test and Fisher exact chi-square test. The Cochran Q test was used to investigate whether there was significant change in osteoporosis incidence during follow-up of patients. Values of p<0.05 were considered statistically significant.

## RESULTS

We did not find any statistically significant difference between the control and patient groups as to age (8.7 vs. 7.0 years, p=0.33) and sex (male and female: 47.8% vs. 63.3% and 52.2% vs. 36.7%, p=0.130, respectively) distribution. There was no N363S polymorphism in either the patient or the control group (Table 1).

The heterozygosity rates of the BclI polymorphism in the control and patient groups were 37% (n=17) and 46.9% (n=23) while the homozygosity rates were 4.3% (n=2) and 12.2% (n=6), respectively. C allele frequencies in the control and patient groups were 69.8% and 59.7% and G allele frequencies 30.2% and 40.3%, respectively. There was no statistically significant difference between the two groups as to allele and polymorphism frequency (Table 1).

### Induction Phase

Because two patients had received the induction phase of ALL therapy at another center, we performed induction phase investigations on 47 patients. In Table 2 the frequencies of adverse events seen during induction are listed. We performed hip MRI for 9 patients and only one of them had grade I avascular necrosis.

Dyspeptic symptoms were more frequent among patients with CG rather than CC genotype (36.4% vs. 5.3%, p=0.018) (Table 3). Depression symptoms were more frequent among polymorphism carriers (CG+GG genotypes) than non-carriers (39.3% vs. 10.5%, p=0.031) (Table 3). There was no significant difference between polymorphism carriers and non-carriers as to hyperglycemia, hypertriglyceridemia, Cushingoid changes, liver function test abnormality, arthralgia, myalgia, muscle weakness, sinus tachycardia, thrombosis, number of FEN attacks, osteopenia and osteoporosis, and hypertension frequency (Table 2).

### Reinduction Phase

Because five patients had been treated at another center, we analyzed the side effects of 44 patients during the reinduction phase. In Table 2 frequencies of adverse events seen during induction are listed. Nine patients were investigated by hip MRI for avascular necrosis. Three patients had osteonecrosis and two of them underwent bilateral hip joint replacement operations. At the end of reinduction we investigated 34 patients based on BMD for osteoporosis. Eight (23.5%) patients had no osteopenia or osteoporosis, but the corrected (for age) Z-scores of 14/34 patients (41.2%) were between -1 and -2.5. Twelve of 34 patients (35.3%) developed osteoporosis and one of these cases was grade 3 (limiting self-care) according to CTCAE v.4.

Cushingoid changes (54.3% vs. 17.6%, p=0.043), depression symptoms (50.0% vs. 11.8%, p=0.011), and dyspepsia (36.4% vs. 0.0%, p=0.008) were more frequent among patients with the CG genotype than those with the CC genotype (Table 4). Cushingoid changes (48.1% vs. 17.6%, p=0.041), dyspepsia (29.6% vs. 0.0%, p=0.016), and depression symptoms (40.7% vs. 11.8%, p=0.040) were more frequent among polymorphism carriers (CG+GG genotypes) than non-carriers (Table 4). There was no significant difference between polymorphism carriers and non-carriers as to hyperglycemia, hypertriglyceridemia, liver function test abnormality, arthralgia, myalgia, muscle weakness, sinus tachycardia, and thrombosis frequency (Table 4).

In total, 22 patients were investigated regularly for BMD at the time of diagnosis, at the end of induction, and at reinduction. There was no significant change between BMD at diagnosis, at the end of induction, and at the end of reinduction (Table 5).

## DISCUSSION

Some children with leukemia experience the side effects of steroids much more so than others, even in an exaggerated manner. GC receptor polymorphisms (N363S and BclI) may cause a genetic predilection to increased sensitivity to GCs, and their early recognition may help clinicians to modify the steroid doses in future trials. In this study, in which we searched for the presence of GR polymorphisms that yield high sensitivity to GCs, we found that carriers of the BclI polymorphism were more prone to side effects of GCs like Cushingoid changes, dyspepsia, and depression symptoms for the first time in the literature as far as we know.

We found that the N363S polymorphism was absent in our population, consistent with the results in Asian populations in which no N363S polymorphism was found [28]. Likewise, it was recently reported in a Turkish population that the frequency of N363S G allele carriers was 0.5% [29]. In contrast to our results, in Europe, the frequency of carrying at least one G allele of the N363S polymorphism was reported to range from 4% to 9% [26,28], highlighting the ethnic factors in its carriage.

The C allele of the BclI polymorphism is the most frequent allele and this is considered to be the wild type [7]. The frequencies of the C and G alleles of the BclI polymorphism are about 65% and 35%, respectively [7]. We found similar results to those in the literature in the control and patient groups with regard to C and G allele frequency (Table 1).

Marino et al. [24] reported the frequencies of GC side effects during induction and reinduction in children with ALL who were treated according to the Italian BFM ALL 2000 study protocol. During the induction phase they found the frequency of depression as 69.4%, Cushingoid changes as 50.0%, hypertriglyceridemia as 8.3%, hyperglycemia as 22.2%, and hypertension as 5.6%. During the reinduction phase, they found the frequency of depression as 58.3%, Cushingoid changes as 58.3%, hyperglycemia as 22.2%, gastritis as 11.1%, hypertension as 2.7%, and hypertriglyceridemia as 0%. We have encountered some adverse events less frequently (Cushingoid changes, depression symptoms) or more frequently (hypertriglyceridemia, hyperglycemia, and hypertension) than Marino et al. [24]. The differences between our results and those of Marino et al. [24] may be due to ethnic and genetic differences. Both N363S and BclI polymorphisms are known to make patients more prone to the side effects of the GCs [28,29,30,31]. The effects of GR gene mutations and polymorphisms on treatment success and drug resistance were also recently investigated and controversial results were obtained [3,20,21,26]. In our study we did not investigate the effect of the BclI polymorphism on treatment success. Alternatively, we focused on the side effects of GCs and their relation with GR gene polymorphisms.

In the literature there are few studies about the side effects of GCs and GR polymorphisms. In Marino et al.’s [24] study no relationship was found between the polymorphisms (N363S and BclI) and appetite, weight gain, Cushingoid changes, depression, anxiety, emotional lability, neuromuscular weakness, myalgia, or severity of infections in pediatric ALL patients during the induction and reinduction periods. Eipel et al. [26] found that the BclI polymorphism was not associated with hepatotoxicity, glucose metabolism abnormalities, central nervous system or behavioral abnormalities, or hypertension. We found no relation between Cushingoid changes and the BclI polymorphism during the induction phase, but a more frequent Cushingoid appearance was seen in patients with CG+GG than in patients with the CC genotype during the reinduction phase (Table 3), in contrast to the results of Marino et al. [24]. In their study Eipel et al. [26] did not analyze Cushingoid appearance as a side effect. Other studies [32,33] found higher levels of subcutaneous fat tissue in BclI polymorphism carriers than in non-carriers. Since Cushingoid changes are related to changes in the distribution of subcutaneous fat tissue, we think that our findings support these findings of previous studies [32,33]. During the reinduction period we used high-dose dexamethasone, which is more potent than prednisolone [23]. The discrepancy in the relationship between BclI polymorphism and Cushingoid appearance during the induction period while the opposite was observed during the reinduction phase may be due to the potency of dexamethasone.

We found that dyspepsia was more frequent in BclI polymorphism carriers than non-carriers during both phases (Tables 3 and 4). We could not find any information about the relationship between GR polymorphisms and dyspepsia or gastrointestinal disorders in the English literature. Therefore, our study seems to be the first study in the literature to report on this aspect, as well.

In our study, depression symptoms were more frequent in BclI polymorphism carriers during both phases (Tables 3 and 4). GCs are known to be related to adverse psychiatric side effects during pediatric ALL treatment [33,34]. Felder-Puig et al. [23] and Eipel et al. [26] found no relation between the BclI polymorphism and adverse psychiatric side effects in pediatric ALL patients. On the other hand, there are reports showing that the BclI polymorphism was more frequent in patients with major depression [11,12]. Therefore, our results are consistent with those in the literature [11,12].

Osteopenia and osteoporosis were more frequent in the CG and GG than in the CC genotype during the two phases, although the difference was not significant (Tables 3 and 4). Similarly, te Winkel et al. [25] reported no relationship between the BclI polymorphism and BMD in 69 pediatric ALL patients.

In conclusion, in our sample, the adequacy of which was confirmed by power analysis, we found that the N363S polymorphism was absent and the BclI polymorphism prevailed, and patients who were carriers of the BclI polymorphism were more prone to side effects of GCs like Cushingoid changes, dyspepsia, and depression symptoms. As far as we know, our study is the first to determine any relation between the BclI polymorphism and symptoms of depression and dyspepsia as side effects of GCs in childhood. The absence of the N363S polymorphism in our study sample in contrast with reports from other countries also highlights the variabilities of side effects of GCs in patients from different ethnic and geographic regions.

In our study, a larger number of patients with definite side effects (including those with sinus tachycardia and osteopenia/osteoporosis) and those consuming standard types of steroids could have provided further information about the relation between side effects and the BclI polymorphism during ALL treatment, which was the main limitation of our study. However, despite these limitations, this pilot study draws attention to the role of the BclI polymorphism on the development of particularly the aforementioned side effects during ALL treatment in Turkey and emphasizes that its role should be validated in further ethnic, regional, and global studies while designing individual treatment modalities.

## Figures and Tables

**Table 1:**
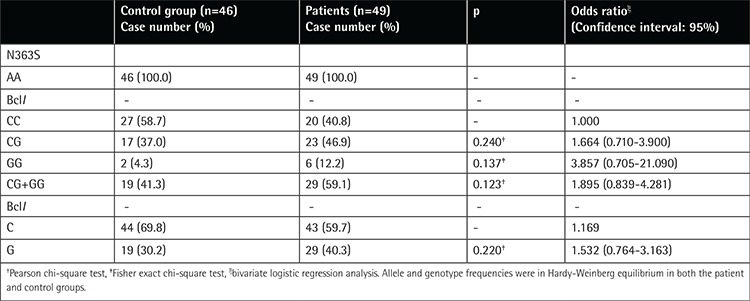
Distribution of N363S and BclI polymorphisms in control and patient groups.

**Table 2:**
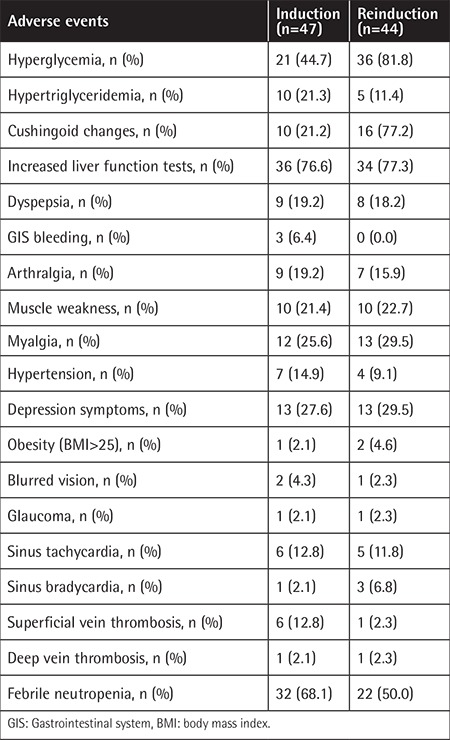
Frequencies of adverse events seen during the induction and reinduction phases.

**Table 3:**
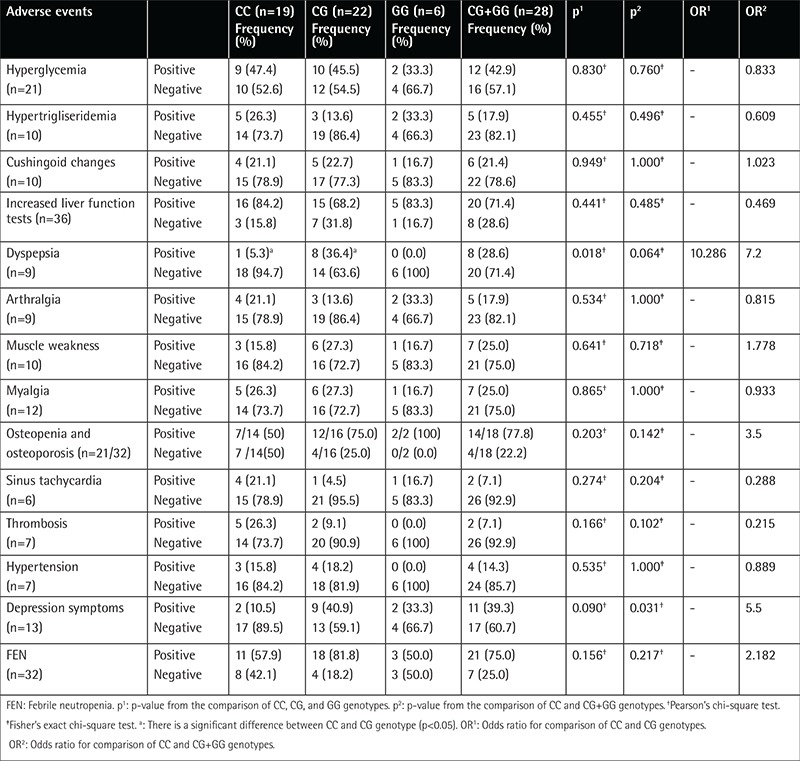
Side effect distributions of patient group during induction phase according to genotypes of BclI polymorphisms.

**Table 4:**
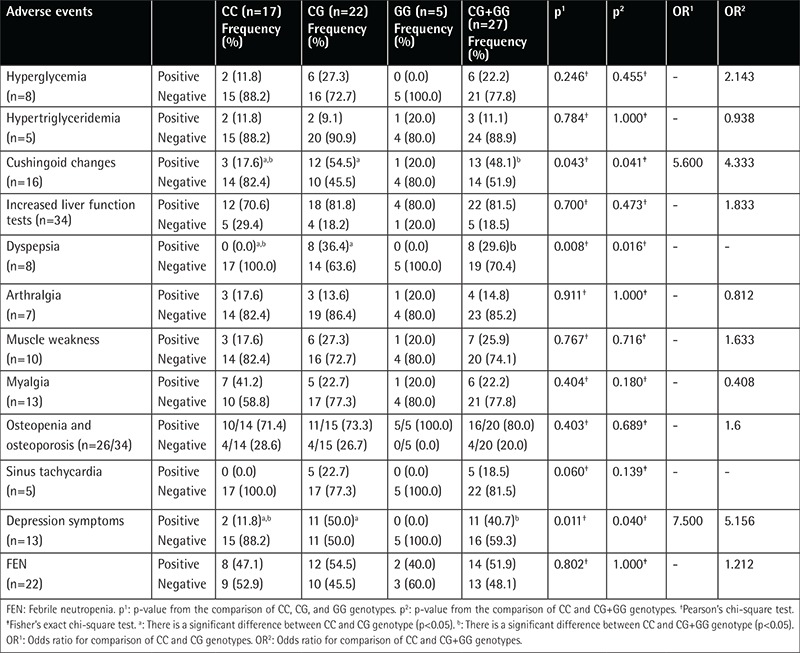
Side effect distributions of patient group during reinduction phase according to genotypes of BclI polymorphism

**Table 5:**
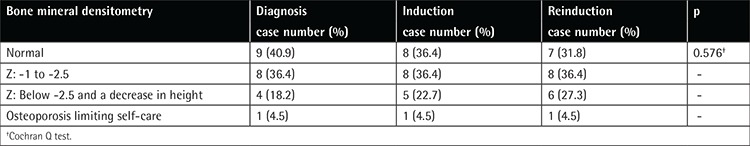
Distribution of bone mineral densitometry results of the patient group.

## References

[1] Yuksel-Soycan L, Aydogan G, Tanyeli A, Timur C, Erbay A, Oniz H, Patiroglu G, Yesilipek A, Soker M, Vural S, Pekun F, Bor O, Sarper N, Cetingul N, Guler E, Polat A, Biner B, Caglar K, Goksan B, Gedikoglu G (2006). BFM-TR ALL 2000: First Turkish multicentric study in the treatment of pediatric acute lymphoblastic leukemia. Pediatr Blood Cancer.

[2] Pui CH, Boyett JM, Rivera GK, Hancock ML, Sandlund JT, Ribeiro RC, Rubnitz JE, Behm FG, Raimondi SC, Gajjar A, Razzouk B, Campana D, Kun LE, Relling MV, Evans WE (2000). Long-term results of total therapy studies 11, 12 and 13A for childhood acute lymphoblastic leukemia at St Jude Children’s Hospital. Leukemia.

[3] Tissing WJ, Meijerink JP, den Boer ML, Brinkhof B, van Rossum EF, van Wering ER, Koper JW, Sonneveld P, Pieters R (2005). Genetic variations in the glucocorticoid receptor gene are not related to glucocorticoid resistance in childhood acute lymphoblastic leukemia. Clin Cancer Res.

[4] Koper JW, Stolk RP, de Lange P, Huizenga NA, Molijn GJ, Pols HA, Grobbee DE, Karl M, de Jong FH, Brinkmann AO, Lamberts SW (1997). Lack of association between five polymorphisms in human glucocorticoid receptor gene and glucocorticoid resistance. Hum Genet.

[5] Huizenga NA, Koper JW, De Lange P, Pols HA, Stolk RP, Burger H, Grobbee DE, Brinkmann AO, De Jong FH, Lamberts SW (1998). A polymorphism in the glucocorticoid receptor gene may be associated with and increased sensitivity to glucocorticoids in vivo. J Clin Endocrinol Metab.

[6] Cuzzoni E, De Ludicibus S, Bartoli F, Ventura A, Decorti G (2012). Association between BclI polymorphism in the NR3C1 gene and in vitro individual variations in lymphocyte-responses to methylprednisolone. Br J Clin Pharmacol.

[7] van Rossum EF, Koper JW, van den Beld AW, Uitterlinden AG, Arp P, Ester W, Janssen JA, Brinkmann AO, de Jong FH, Grobbee DE, Pols HA, Lamberts SW (2003). Identification of BclI polymorphism in the glucocorticoid receptor gene: association with sensitivity to glucocorticoid in vivo, and body mass index. Clin Endocrinol (Oxf).

[8] Derijk RH, de Kloet ER (2008). Corticosteroid receptor polymorphisms: determinants of vulnerability and resilience. Eur J Pharmacol.

[9] Lin RC, Wang XL, Morris BJ (2003). Association of coronary artery disease with glucocorticoid receptor N363S variant. hypertension.

[10] Di Blasio AM, van Rossum EF, Maestrini S, Berselli ME, Tagliaferri M, Podestà F, Koper JW, Liuzzi A, Lamberts SW (2003). The relation between two polymorphisms in the glucocorticoid receptor gene and body mass index, blood pressure and cholesterol in obese patients. Clin Endocrinol (Oxf).

[11] Rossum EF, Binder EB, Majer M, Koper JW, Ising M, Modell S, Salyakina D, Lamberts SW, Holsboer F (2006). Polymorphisms of the glucocorticoid receptor gene and major depression. Biol Psychiatry.

[12] Krishnamurthy P, Romagni P, Torvik S, Gold PW, Charney DS, Detera-Wadleigh S, Cizza G; P (2008). O.W.E.R. (Premenopausal, Osteoporosis Women, Alendronate, Depression) Study Group. Glucocorticoid receptor gene polymorphisms premenopausal women with major depression. Horm Metab Res.

[13] Zobel A, Jessen F, von Widdern O, Schuhmacher A, Höfels S, Metten M, Rietschel M, Scheef L, Block W, Becker T, Schild HH, Maier W, Schwab SG (2008). Unipolar depression and hippocampal volume: impact of DNA sequence variants of the glucocorticoid receptor gene. Am J Med Genet B Neuropsychiatr Genet.

[14] Savic D, Knezevic G, Damjanovic S, Antic J, Matic G (2014). GR gene BclI polymorphysm changes the path, but not the level, of dexamethasone-induced cortisol suppression. J Affect Disord.

[15] Lauten M, Stanulla M, Zimmermann M, Welte K, Riehm H, Schrappe M (2001). Clinical outcome of patients with childhood acute lymphoblastic leukemia and an initial leukemic blast count less than 1000 per microliter. Klin Padiatr.

[16] Schrappe M, Reiter A, Zimmermann M, Harbott J, Ludwig WD, Henze G, Gadner H, Odenwald E, Riehm H (2000). Long term results of four consecutive trials in childhood ALL performed by the ALL-BFM study group from 1981 to 1995. Berlin-Frankfurt-Münster. Leukemia.

[17] Hillmann AG, Ramdas J, Multanen K, Norman MR, Harmon JM (2000). Glucocorticoid receptor gene mutations in leukemic cells acquired in vitro and in vivo. Cancer Res.

[18] Catts VS, Farnsworth ML, Haber M, Norris MD, Lutze-Mann LH, Lock RB (2001). High level resistance to glucocorticoids, associated with a dysfunctional glucocorticoid receptor, in childhood acute lymphoblastic leukemia cells selected for methotrexate resistance. Leukemia.

[19] Irving JA, Minto L, Bailey S, Hall AG (2005). Loss of heterozygosity and somatic mutations of the glucocorticoid receptor gene are rarely found at relapse in pediatric acute lymphoblastic leukemia but may occur in a subpopulation early in the disease course. Cancer Res.

[20] Labuda M, Gahier A, Gagné V, Moghrabi A, Sinnett D, Krajinovic M (2010). Polymorphisms in glucocorticoid receptor gene and the outcome of childhood acute lymphoblastic leukemia (ALL). Leuk Res.

[21] Namazi S, Zareifar S, Monabati A, Ansari S, Karimzadeh I (2011). Evaluating the effect of 3 glucocorticoid receptor gene polymorphisms on risk relapse in 100 Iranian children with acute lymphoblastic leukemia: a case-control study. Clin Ther.

[22] Eipel OT, Németh K, Török D, Csordás K, Hegyi M, Ponyi A, Ferenczy A, Erdélyi DJ, Csóka M, Kovács GT (2013). The glucocorticoid receptor gene polymorphism N363S predisposes to severe toxic side effects during pediatric acute lymphoblastic leukemia (ALL) therapy. Int J Hematol.

[23] Felder-Puig R, Scherzer C, Baumgartner M, Ortner M, Aschenbrenner C, Bieglmayer C, Voigtländer T, Panzer-Grümayer ER, Tissing WJ, Koper JW, Steinberger K, Nasel C, Gadner H, Topf R, Dworzak M (2007). Glucocorticoids in the treatment of children with acute lymphoblastic leukemia and Hodgkin’s disease: a pilot study on the adverse psychological reactions and possible associations with neurobiological, endocrine and genetic markers. Clin Cancer Res.

[24] Marino S, Verzegnassi F, Tamaro P, Stocco G, Bartoli F, Decorti G, Rabusin M (2009). Response to glucocorticoids and toxicity in childhood acute lymphoblastic leukemia: role of polymorphisms of genes involved in glucocorticoid response. Pediatr Blood Cancer.

[25] te Winkel ML, van Beek RD, Uitterlinden AG, Hop WC, Pieters R (2010). Pharmacogenetic risk factors for altered bone mineral density and body composition in pediatric acute lymphoblastic leukemia. Haematologica.

[26] Eipel O, Hegyi M, Csordas K, Nemeth K, Luczay A, Török D, Csoka M, Erdeyi D, Kovacs G (2016). Some GCR polymorphisms (N363S, ER22/23K, and Bcl-1) may influence steroid-induced toxicities and survival rates in children with ALL. J Ped Hematol Oncol.

[27] no author (2010). National Institutes of Health. Common Terminology Criteria for Adverse Events (CTCAE) Version 4.0. Washington, U.S. Department of Health and Human Services, National Institutes of Health. National Cancer Institute.

[28] Koper JW, van Rossum EF (2014). Glucocorticoid receptor polymorphisms and haplotypes and their expression in health and disease. Steroids.

[29] Kaya Z, Caglayan S, Akkiprik M, Aral C, Ozisik G, Ozata M, Ozer A (2016). Impact of glucocorticoid receptor gene (NR3C1) polymorphisms in Turkish patients with metabolic syndrome. J Endocrinol Invest.

[30] Roussel R, Reis AF, Dubois-Laforgue D, Bellanné-Chantelot C, Timsit J, Velho G (2003). The N363S polymorphism in the glucocorticoid receptor gene is associated with overweight in subjects with type 2 diabetes mellitus. Clin Endocrinol (Oxf).

[31] Manenschijn L, Lamberts SW, van Rossum EF (2009). Clinical features associated with glucocorticoid receptor polymorphisms. Ann N Y Acad Sci.

[32] Tremblay A, Bouchard L, Bouchard C, Després JP, Drapeau V, Pérusse L (2003). Long-term adiposity changes are related to glucocorticoid receptor polymorphism in young females. J Clin Endocrinol Metab.

[33] Harris JC, Carel CA, Rosenberg LA, Joshi P, Leventhal BG (1986). Intermittent high dose corticosteroid treatment in childhood cancer: behavioral and emotional consequences. J Am Acad Child Psychiatry.

[34] Drigan R, Spirito A, Gelber RD (1992). Behavioral effects of corticosteroids in children with acute lymphoblastic leukemia. Med Pediatr Oncol.

